# Remaining Useful Life Estimation of Aircraft Engines Using a Joint Deep Learning Model Based on TCNN and Transformer

**DOI:** 10.1155/2021/5185938

**Published:** 2021-11-24

**Authors:** Hai-Kun Wang, Yi Cheng, Ke Song

**Affiliations:** ^1^School of Artificial Intelligence, Chongqing University of Technology, Chongqing 40400, China; ^2^Chongqing Industrial Big Data Innovation Center Co., Ltd., Chongqing 40400, China

## Abstract

The remaining useful life estimation is a key technology in prognostics and health management (PHM) systems for a new generation of aircraft engines. With the increase in massive monitoring data, it brings new opportunities to improve the prediction from the perspective of deep learning. Therefore, we propose a novel joint deep learning architecture that is composed of two main parts: the transformer encoder, which uses scaled dot-product attention to extract dependencies across distances in time series, and the temporal convolution neural network (TCNN), which is constructed to fix the insensitivity of the self-attention mechanism to local features. Both parts are jointly trained within a regression module, which implies that the proposed approach differs from traditional ensemble learning models. It is applied on the Commercial Modular Aero-Propulsion System Simulation (C-MAPSS) dataset from the Prognostics Center of Excellence at NASA Ames, and satisfactory results are obtained, especially under complex working conditions.

## 1. Introduction

With the progress of industrial technology and the upgrading of global industry, the demand for safe and reliable products and equipment in all fields is gradually increasing. Prognostics and health management (PHM) [1] has received considerable attention. Remaining useful life (RUL) prediction is a core task in PHM. Generally, the RUL of the system is defined as “the length from the current time to the end of the useful life” [2]. The main purpose of RUL prediction is to monitor the health status of system equipment, so that system maintenance personnel can know the current operating status of system equipment in real time, implement condition-based predictive maintenance, and reduce system maintenance costs while avoiding unexpected failures of the system [3].

In the literature, the basic algorithms for predicting RUL can be divided into two categories, i.e., physical model-based approaches and data-driven model-based approaches [4]. The first approaches describe the degradation stage of a system by constructing mathematical models on the basis of the failure mechanisms or the first principle of damage [3]. The physical model established with an in-depth understanding of failure modes and effective estimation of model parameters can provide accurate RUL estimation, e.g., the Paris-Erdogan (PE) model to describe crack growth [5, 6] and the Norton law to describe the creep evolution of turbines [7, 8]. However, physical model-based approaches have two problems that are difficult to solve. One is that the established physical model is difficult to be directly applied to other systems; the other is that the establishment of an efficient physical model requires complex prior knowledge. Based on these limitations, data-driven approaches are increasingly being valued. Among them, stochastic model-based approaches are the first to bear the brunt. The gamma process model has been used in RUL prediction tasks, and there is much additional research [9]; however, gamma process models are only effective in describing monotonic processes, because noise must follow a gamma distribution. Huang et al. [10] proposed a nonlinear heterogeneous Wiener process model with adaptive drift to characterize degradation trajectories, but the Wiener process (including some other stochastic processes) is based on the assumption of the Markov property. This assumption does not always work in applications.

Benefitting from the advent of the industrial big data era, the scale of system status monitoring data collected by sensors has continued to grow. The in-depth architecture-based method provides better generalization capabilities and scalability and does not require special professional prior knowledge. Deep learning has achieved great success in various fields [11–13]. In recent years, research on deep learning in RUL prediction has also made progress.

Recent researchers have established many models based on deep architecture for RUL prediction tasks. Bhattacharya et al. [14] proposed using the moth flame optimization (MFO) algorithm for feature selection and used the features in a DNN. Their excellent results on the battery RUL prediction task proved the effectiveness of the deep architecture. Badu et al. [15] applied a CNN to RUL prediction for the first time, applied convolution and pooling filters to multichannel sensor data along the time dimension, and achieved competitive results. Li et al. [16] extracted the time-frequency domain features from the degradation data of rolling bearings and used the multiscale time-frequency domain features as the input of a CNN to develop an intelligent bearing RUL prediction method. However, system status monitoring data are often time series data, and CNNs can only extract local features and lack the ability to capture and learn the long-term dependencies in the data. A deep belief network (DBN) is a probabilistic generative model that is composed of multiple restricted Boltzmann machines (RBMs). Zhang et al. [17] proposed a multiobjective deep belief network ensemble (MODBNE) model. MODBNE regards each DBN as a conflicting object and applies a multiobjective evolutionary algorithm based on the basic DBN training method to produce an ensemble model composed of multiple DBNs. Zemouri and Gouriveau [18] and Zhang et al. [19] proposed an RUL prediction model based on a recurrent neural network (RNN) [20]. An RNN can process sequence data, but it encounters difficulties in processing long sequences of data due to the vanishing gradient problem. An LSTM is an improved approach derived from an RNN. Based on an RNN, a gating mechanism is introduced to control the information flow in the memory unit, which solves the vanishing gradient problem in RNNs and makes it possible to learn the dependencies with a relatively long span. References [21, 22] used a long short-term memory (LSTM) network to predict the RUL of turbofan engines. Zhang et al. [23] used an LSTM to predict the RUL of lithium batteries, and their results also proved the effectiveness of the method based on this model. Some researchers have also made improvements to the vanilla LSTM to improve the performance of RUL prediction. Li et al. [24] optimized the connection between the input gate and the forget gate, strengthened the focus on historical data, and improved the accuracy of predicting the RUL of lithium batteries. Considering that the optimization of hyperparameters is always a difficult and time-costly task for deep models, Agrawal et al. [25] proposed optimizing an LSTM with a genetic algorithm (GA) to be able to autonomously predict the given hyperparameters and improve the consistency of predictions. The stacking of different models has also become a way to improve prediction performance. Al-Dulaimi et al. [26] proposed a parallel network composed of an LSTM and a CNN to predict the RUL of an engine and achieved excellent results. Bi-LSTM consists of two opposite LSTM networks and can input data in both forward and backward directions, which further improves the data processing capability of LSTMs. The literatures [27, 28] have used Bi-LSTM-based approaches in the RUL prediction task. Jiang et al. [29] and Remadna et al. [30] further combined the Bi-LSTM and CNN to develop a new fusion model. Inspired by the idea of an encoder-decoder, Liu et al. [31] developed a new learning-based encoder-decoder model based on the LSTM and CNN to predict RUL. Different from Jiang et al. [29], Liu et al. [31] used series LSTM and CNN as an encoder and then used a fully connected neural network as a decoder. Some recent studies are summarized in Table 1 for reference.

CNN-based prediction approaches perform well in local context feature extraction, but they cannot capture long-term dependency. RNN-based prediction approaches are limited by the recurrent mode, which fundamentally limits their computing speed [36]. Especially, when processing long series of data, the time cost of both training and inference will increase, so it is difficult to realize real-time prediction. The transformer network proposed by Vaswani et al. [37] was first applied in the natural language processing (NLP) field, and since then, it has been successfully applied in various fields [38, 39].

The transformer is completely based on the self-attention mechanism. Compared with the sequential input of the RNN, the transformer inputs the whole sequence at one time and uses scaled dot-product attention to capture cross-distance dependencies. The interval of historical data will not become an obstacle, which provides the transformer with more potential than recurrent networks in obtaining long-term dependencies. On the other hand, the special self-attention mechanism can realize parallel computing, and the computing cost will not be the upper limit of the model. Based on this, this paper uses the transformer encoder for sequence modelling to predict the RUL. Considering that the self-attention mechanism is not particularly sensitive to the local context and that the sensor data often have a strong local correlation, we propose to use a convolutional neural network (CNN) to extract local context information.

In this work, we used three different convolution-based neural networks to extract the local features of the input time series data as a supplement to the transformer encoder. Two of them are deep residual networks (ResNets) [40] and densely connected convolutional networks (DenseNets) [41], which have achieved great success in the field of computer vision [42, 43]. The other is called the temporal convolution neural network (TCNN) in this work, which is different from ResNet and other networks designed for image feature extraction. TCNN uses 1D-convolution instead of 2D-convolution. The reason for adopting this approach is to ensure that the sensor values in each time step of the multisensor data sequence are regarded as a whole, because they jointly explain the state of the system in current time step. In our proposed joint deep learning model, the CNN module and the transformer module extract the features of the input data, and then these features are fed into the regression module together. We provide the feature recalibration mechanism (FRM) in the regression module to solve the problem of unequal output feature levels of multiple models.

The proposed joint learning model provides a new scheme to predict RUL, which can flexibly extract the required features through the collocation of different models (two models are used in this experiment, but they are extensible under the premise of meeting the time cost), and then recalibrate the output of different models using FRM. Experiments on the C-MAPSS dataset show that our model has a significant performance improvement compared with previous work under complex operating conditions and failure modes.

This work's main contributions are as follows:

1. We proposed a 1D convolution network called TCNN in this work to emphasize the contribution of the local context of time series data. The reason for proposing TCNN is that although the dot-product self-attention extracts high-level features at each time step regardless of distance, its own characteristics preclude it from giving extra attention to local contexts. The local context is particularly important for studying the degradation patterns in multisensor sequence data.
2. We applied FRM in the regression module to further process the features extracted by the submodels instead of directly feeding them to the fully connected layer. This function gives the model the ability to automatically evaluate the importance of features from different submodels, which makes our joint learning model different from general hybrid models.

The overall organization of the paper is as follows.  Section 2 introduces the structure of the proposed model.  Section 3 describes the dataset and preprocessing method.  Section 4 presents the experimental results and analysis. The conclusions and future perspectives of the work are shown in Section 5.

## 2. Methodology

A joint deep learning model combined with a transformer encoder and CNN is constructed to predict the RUL. In this section, we first introduce the transformer encoder and then introduce the structure of CNNs used in this paper. Finally, we describe the regression module used to output the predicted RUL. Parameter settings are given in Sections 2.4 and 4.2.

### 2.1. Transformer Encoder

The transformer [37] is composed of an encoder and decoder. It is a feature extractor based on the self-attention mechanism. Different from RNN, LSTM [44], and other recurrent neural networks, the transformer accepts the whole time series at the same time and completely depends on the attention mechanism to draw global dependencies between input and output. In addition, the multihead mechanism allows the model to jointly attend to information from different representation subspaces at different time steps.

With the continuous use of the system, its performance will gradually degenerate until the threshold of failure. The degradation evolution of the system can be indirectly reflected from the collected sensor data. In this work, the encoder part of the transformer is used as a feature extractor to extract potential degraded features.

**Figure 1:**
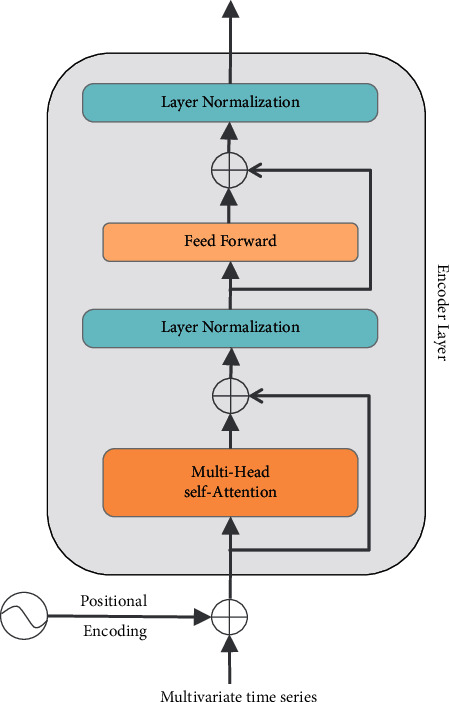
The structure of the transformer encoder layer.

The transformer encoder is composed of a stack of several identical encoder layers, and each encoder layer consists of two sublayers: a self-attention sublayer and a fully connected feedforward sublayer. Each sublayer is followed by layer normalization; in addition, the residual connection is applied to the input and output of each sublayer. The input to the transformer encoder is a multivariate time series, which is represented as *X* ∈ *ℝ*^*k*×*d*^, where *k* is the length of the time series, and *d* is the dimension of features. The output dimension of each transformer encoder layer is still *k* × *d*. The structure of the transformer encoder layer for RUL prediction is shown in Figure 1.

#### 2.1.1. Positional Encoding

Before the transformer encoder, for the model to take advantage of the sequence order, we must inject some information about the relative or absolute position of the time point in the sequence. Following the work in [37], sine and cosine functions of different frequencies are used for position encoding:

(1) $$\begin{array}{c} {\text{PE}}_{\left(\text{pos},2i\right)}=\sin\left({\frac{\text{pos}}{10000}}^{2i/d}\right),\\ {\text{PE}}_{\left(\text{pos},2i+1\right)}=\cos\left({\frac{\text{pos}}{10000}}^{2i/d}\right), \end{array}$$

where pos is the position of a time point, and *i* is the index of the feature. PE is a position embedding matrix, whose shape depends on the input sequence. Using this function, the model would be allowed to easily learn to attend by relative positions. Then, we only need to add the position embedding matrix and the input sequence. Note that the position embedding matrix is given and does not need to be learned in training in this work.

#### 2.1.2. Multihead Self-Attention

Multihead self-attention can be interpreted as applying multiple self-attention mechanisms called the scaled dot-product attention function. The output of each self-attention function is called a head. Scaled dot-product attention can be depicted as mapping a query and a set of key-value pairs to output, where the queries, keys, and values are derived from the linear mapping of the representation vector at a time point. The output is computed as a weighted sum of the values, where the weight assigned to each value is computed by a compatibility function of the query with the corresponding key. In practice, all queries, keys, and values, which are packaged as matrices *Q*, *K*, and *V*, respectively, are computed in parallel by matrix operation. For simplicity, it is still assumed that the input of the multihead self-attention module is multivariate time series *X* ∈ *ℝ*^*k*×*d*^. Then, the formula for calculating *Q*, *K*, and *V* can be expressed by

(2) $$\begin{array}{c} Q_{j}=XW_{j}^{Q},\\ K_{j}=XW_{j}^{K},\\ V_{j}=XW_{j}^{V}, \end{array}$$

where *W*_*j*_^*Q*^ ∈ *ℝ*^*d*×*d*_*k*_^, *W*_*j*_^*K*^ ∈ *ℝ*^*d*×*d*_*k*_^, and *W*_*j*_^*V*^ ∈ *ℝ*^*d*×*d*_*v*_^ are the learned weight matrices used to calculate the matrices *Q*_*j*_, *K*_*j*_ and *V*_*j*_ of head *j*, respectively, *d*_*k*_ is the size of *W*_*j*_^*Q*^ and *W*_*j*_^*K*^, and *d*_*v*_ is the size of *W*_*j*_^*V*^. Then, apply the scaled dot-product attention function to *Q*_*j*_, *K*_*j*_, *V*_*j*_:

(3) $$\begin{array}{c} \mathrm{Attention}\;\left(Q_{j},K_{j},V_{j}\right)=\mathrm{softmax}\;\left(\frac{Q_{j}K_{j}^{T}}{\sqrt{d_{k}}}\right)V_{j}. \end{array}$$

Finally, the output of the multihead self-attention sublayer can be obtained according to the following formula:

(4) $$\begin{array}{c} \text{MultiHead}\left(Q,K,V\right)=\text{Concat}\left({\text{head}}_{1},\ldots ,{\text{head}}_{h}\right)W^{O},\\ \mathrm{Where}\;{\text{head}}_{j}=\text{Attention}\left(Q_{j},K_{j},V_{j}\right), \end{array}$$

where *h* is the number of heads, and *W*^*O*^ ∈ *ℝ*^*hd*_*v*_×*d*^ is the parameter matrix that maps the multiple heads back to *ℝ*^*k*×*d*^. In this work, we set *d*_*k*_=*d*_*v*_=16, and *h*=8. Three transformer encoder layers are created.

#### 2.1.3. Feed-Forward Network

A fully connected feed-forward network (FFN) consists of two linear transformations with a rectified linear unit activation function (ReLU) [45] in between, which is applied to each encoder layer separately and identically.

(5) $$\begin{array}{c} \text{FFN}\left(x\right)=\max\left(0,xW_{1}+b_{1}\right)W_{2}+b_{2}. \end{array}$$

### 2.2. CNNs for Local Feature Extraction

One of the main advantages of the transformer is that it uses the attention mechanism to model global dependencies among nodes in input data, but it does not pay special attention to local dependencies. However, for multivariate time series, especially for the multivariate industrial sensor data of interest in this paper, there is often a strong correlation between adjacent time steps. Naturally, we propose to use a convolution-based network as a local feature extractor of the input data as a supplement to the transformer.

In this work, we present three convolution-based neural network architectures as local feature extractors. They are the TCNN proposed in this paper and the classical networks: deep residual network (ResNet) and densely connected convolutional network (DenseNet).

**Figure 2:**
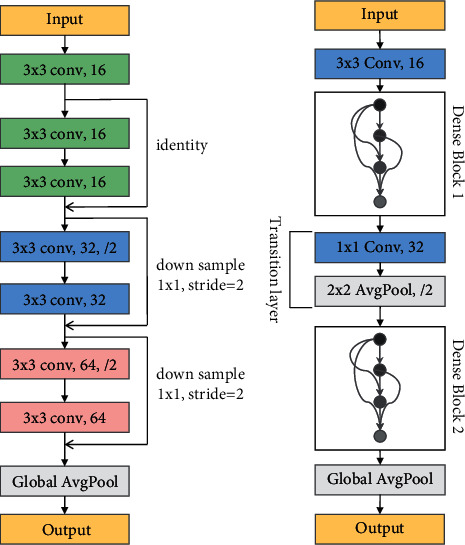
The classic CNNs used in this paper. (a) DenseNet. (b) ResNet.

ResNet was first proposed by He et al. [40], which was developed to tackle the issue of degradation and vanishing gradients, and won 1st place in the ILSVRC competition in 2015. This network has achieved great success in the field of computer vision. A light ResNet used in this work is shown in Figure 2(a). Each convolution layer is followed by batch normalization and an ReLU (not shown in the figure). For a complete explanation of ResNet, refer to [40].

DenseNet [41] shows excellent performance by encouraging feature reuse and significantly reduces the number of parameters and computational cost. The way to build DenseNet architecture is to make further use of features on the basis of shortcut connections proposed by ResNet, that is, to establish a connection between all pairs of layers in the network, so that the layer can obtain the features of all preceding feature maps. A light DenseNet with structures “bottleneck” and “compression” used in this work is shown in Figure 2(b). For a complete explanation of DenseNet, refer to [41].

**Figure 3:**
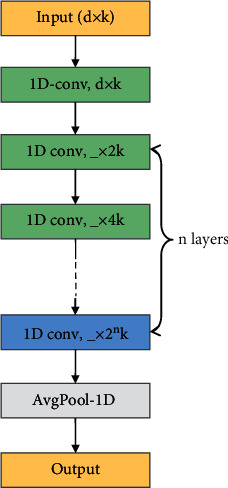
The structure of TCNN.

TCNN is different. ResNet or other traditional CNN networks are designed in the field of computer vision. They all carry out the 2D convolution operation, and all convolution kernel sizes are usually design parameters such as 3 × 3 or 5 × 5. However, the sensor values in each time step of the multisensor data sequence should be regarded as a whole, because they jointly explain the state of the object in this time step. Therefore, we propose to use 1D convolution instead of 2D convolution to obtain better RUL prediction results. The structure of the TCNN we used is described in Figure 3. Each convolution block in Figure 3 actually contains a convolution layer, a batch normalization layer, an activation layer, and a dropout layer in order. The kernel size of all 1D convolution layers is 3, and the padding is 1. The stride of the first 1D convolution layer is 1, and the number of output channels is the same as that of the input. The stride of the other 1D convolution layers is 2, and the number of output channels is twice that of the input. This means that every time the features are halved, the number of channels doubles. Finally, an average pooling operation is performed for each channel. In this work, the activation function is ReLU, the dropout rate is 0.5, and a total of 4 convolution layers were built.

### 2.3. Regression Module

We concatenate the output features of the transformer encoder and CNN (one of TCNN, ResNet, or DenseNet) to form a feature vector *x* ∈ *ℝ*^*m*^ (*m* is the sum of the feature numbers of the transformer encoder and CNN output). To obtain the predicted value of RUL, the usual method is that the feature vector is directly fed to the regression module to complete the regression task. However, the two parts of the features of *x* come from the parallel processing of the input multivariate time series by the transformer encoder and CNN. Usually, we cannot measure the level of the output features of the two modules. Therefore, we apply FRM to *x*. The FRM can be summarized as letting *x* go through a two-layer fully connected network to output a normalized vector *v* with the same dimension as *x* and then taking the Hadamard product of *x* and *v* to obtain the recalibrated *x*. Its mathematical expression is

(6) $$\begin{array}{c} x^{\prime }=x\circ v=x\circ \text{Sigmoid}\left(W_{2}^{\prime }\text{ ReLU}\left(W_{1}^{\prime }x\right)\right), \end{array}$$

where *x*′ is the recalibrated *x*, which is also the vector finally fed to the regression module. *W*_1_′ ∈ *ℝ*^(*m*/16)×*m*^ and *W*_2_′ ∈ *ℝ*^*m*×(*m*/16)^ are both parameter matrices determined in training, and “∘” is the Hadamard product. To obtain *v*, we use two fully connected layers. The first layer uses the ReLU activation function, which helps increase the nonlinearity of the transformation. In addition, the first layer reduces the dimension to 1/16 of the original, which helps reduce the computational consumption. Due to the sigmoid activation function, *v* is normalized to (0,1), which means that, after training, the model can decide whether to give a value in **x** a large weight or a small weight to recalibrate the feature vector **x**.

Finally, *x*′ is fed to a two-layer fully connected network (FCN). In this work, the size of the hidden layer is obtained by the random search algorithm introduced in Section 2.4, and the activation function is ReLU. There is only one output of the output layer, i.e., the predicted RUL.

### 2.4. Loss Function and Hyperparameters

#### 2.4.1. Loss Function

The mean square error (MSE) is used to build the loss function, as shown in the following equation:

(7) $$\begin{array}{c} \text{Loss}=\frac{1}{B}{\left\|y_{\text{pre}}-y_{\text{target}}\right\|}^{2}, \end{array}$$

where *y*_pre_ and *y*_target_ are the predicted output of the proposed model and the established target output, respectively. *B* is the number of units in a minibatch in the training. We use Adam as the optimizer of our model.

#### 2.4.2. Hyperparameter Selection

The hyperparameters of the deep model have a significant impact on the results. Although, in the case of this article, it is feasible to implement manual search and grid search (complete training and testing only takes a few hours), but considering that the application of manual search or grid search to new datasets is a poor choice, we use an easy-to-implement and effective random search algorithm [46] in this work. There are 7 hyperparameters determined by random search, of which only the learning rate is a continuous value, and the rest are discrete values. The number of encoder layers, the number of TCNN layers, and the kernel size of the TCNN are lists with increments of 1, and the last three rows in Table 2 are lists with exponential growth.

### 2.5. Model Complexity

We analyzed the complexity and parameter requirements of the two core components of the transformer (i.e., self-attention and feed-forward network) and the three CNN architectures established in this paper in Table 3. The complexity of CNNs is determined by their convolution operation. When the input sequence is relatively short, the bottleneck of the transformer encoder is the FFN. However, when the dimension of D is not high, the complexity of the self-attention module will dominate with increasing input sequence length. The transformer is a general and flexible architecture, but its disadvantage is that transformer does not introduce a priori knowledge about the input data structure, and its information transmission process completely depends on the similarity measurement of content. This is why we choose to introduce CNN architecture into our joint learning model.

## 3. Dataset and Preprocessing

### 3.1. Data Description

In this work, the Commercial Modular Aero-Propulsion System Simulation (C-MAPSS) [47] dataset was used to support this study, and this dataset was previously reported by the Prognostics CoE at NASA Ames. This dataset is available at https://ti.arc.nasa.gov/tech/dash/groups/pcoe/prognostic-data-repository. The information of the C-MAPSS dataset used is listed in Table 4. C-MAPSS consists of four datasets (from FD001 to FD004), corresponding to different operating conditions and fault mode combinations. Each dataset is further divided into training and test subsets. Datasets consist of multiple multivariate time series, and each dataset can be regarded as an *n* × 26 matrix, where *n* corresponds to the number of time cycles, which contains 26 columns of operation state data. The first column is the engine ID, the second column is the time cycle index, and the third to fifth columns indicate the operating conditions. The remaining variables represent the 21 sensor readings that reflect the engine degradation over time.

The engine runs normally at the start of each time series and develops a fault at some step during the time series. In the training subset, the fault grows in magnitude until system failure. In the test subset, the time series ends at a point prior to the complete system failure. C-MAPSS also provided a vector that records the ground truth of the RUL for the test engines.

### 3.2. Degradation Curve

**Figure 4:**
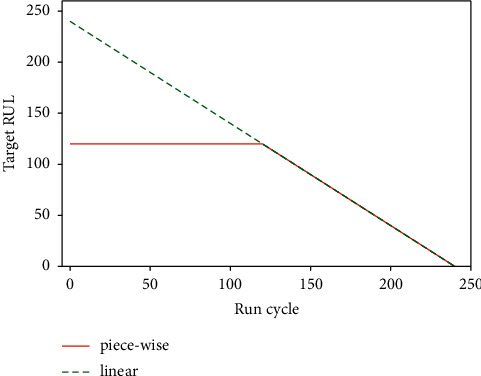
Comparison of linear and piecewise degradation.

The degradation of the system usually begins after a certain period of usage time. In the early stage of system use, it is difficult to accurately predict the RUL, and the predicted RUL will have a large deviation from the actual situation. Such prediction is not of great significance, because the system state is still very healthy at this time. Therefore, a piecewise model was used instead of a linear model to construct a degradation curve. The piecewise model was originally presented in [48], and it has been proven to be an effective method to improve the prediction performance of the model [49, 50]. Specifically, the previous stage of the degradation curve is set to a constant and then begins to degenerate linearly. In this work, the constant value is set to 120. The degradation curve is shown in Figure 4.

### 3.3. Data Preparation

The FD002 and FD004 datasets have six different operating conditions. In this work, one-hot encoding is used to encode six different operating conditions and then replace the data from Columns 2 to 4 of the dataset.

The C-MAPSS dataset provides the readings of 21 sensors at each sampling point, and the details of each sensor are given in the literature [47]. However, not every sensor provides useful degradation information. For example, the measurement results of some sensors do not show a correlation with time in the whole life cycle of the unit. According to the research results in the literature [51], we selected the outputs of 14 sensors from 21 sensors to build the training samples and test samples of the deep model.

To eliminate the influence of different scales of sensor readings, it is necessary to normalize the data of each sensor to be within the range of (0,1) according to equation (8) before any training and testing.

(8) $$\begin{array}{c} \text{Norm}\left(X^{f}\right)=\frac{\left(X^{f}-X_{\min}^{f}\right)}{X_{\max}^{f}-X_{\min}^{f}+\alpha }, \end{array}$$

where *X*^*f*^ represents the readings of sensor *f* over the whole time cycle. We applied a trick here. Because the degradation curve is built by the piecewise model, there will be a large number of training samples with a label value of 120, which is a kind of sample imbalance. A sigmoid function is used in the RUL output neuron. To avoid too many label values being unreachable, we add a scaling value *α*=1 to the denominator in equation (8), so that the maximum value of normalized data is slightly less than 1.

The sliding time window method is applied to build training samples. For the proposed prediction model, we hope that the time series as training samples can be as long as possible to obtain more context information. However, because of the minimum sampling length of test engines, as seen in Table 4, the size of the corresponding sliding windows of FD001 to FD004 is set to 31, 21, 38, and 19.

## 4. Experiments & Results

In this section, we first briefly introduce the evaluation metrics. Then, we present the experimental results of our joint models on C-MAPSS to evaluate the performance for RUL prediction and analyze the experimental results. Finally, we compared the joint model with previous works.

### 4.1. Performance Evaluation

**Figure 5:**
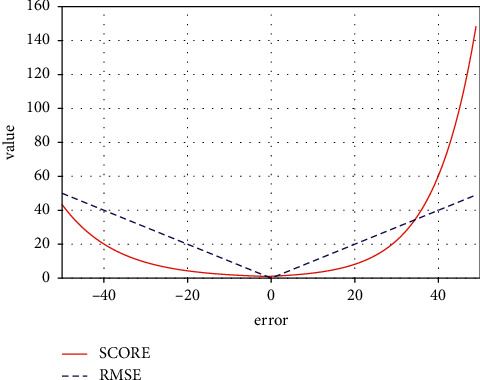
Score function and RMSE.

To measure the prediction performance of the proposed joint model, two evaluation functions are used: scoring function [47] and root mean square error (RMSE) [52]. The formula is as follows:

(9) $$\begin{array}{c} \text{RMSE}=\sqrt{\frac{1}{N}\overset{N}{\underset{i=1}{\sum }}e_{i}^{2}}, \end{array}$$

(10) $$\begin{array}{c} \text{Score}=\left\{\begin{array}{cc} \overset{N}{\underset{i=1}{\sum }}\left(e^{-e_{i}/13}-1\right) & \mathrm{for}\;e_{i}<0\\ \overset{N}{\underset{i=1}{\sum }}\left(e^{e_{i}/10}-1\right) & \mathrm{for}\;e_{i}\geq 0 \end{array}\right., \end{array}$$

where *N* is the number of engines and *e*_*i*_=RUL_*i*_^(prediction)^ − RUL_*i*_^(target)^. RMSE is a common metric used to evaluate the error of prediction values. Equation (10) shows that the scoring function has a greater penalty for overestimated RUL, because overestimated RUL will lead to unexpected engine failure, while underestimated RUL will only lead to early maintenance. Obviously, an underestimated RUL will cause less damage and is more likely to be accepted. The combination of RMSE and score can evaluate the performance of the model more effectively. The comparison of the metrics is shown in Figure 5.

### 4.2. Hyperparameter Selection

**Figure 6:**
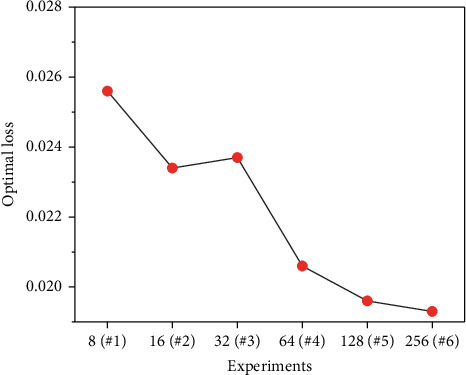
The optimal loss of each of the 6 experiments.

We used a random search algorithm to determine the 7 hyperparameters described in Section 2.4. A total of 6 experiments were implemented. As shown in Figure 6, each scale on the abscissa represents an experiment. The scale value represents how many independently identically distributed random searches and trainings have been performed in this experiment. Note that there is no overlap in each experiment, which means that a total of 504 trainings were performed instead of 256. The hyperparameter values chosen by the random search algorithm are shown in Table 5.

### 4.3. RUL Prediction of Joint Models

#### 4.3.1. Prediction Results

**Figure 7:**
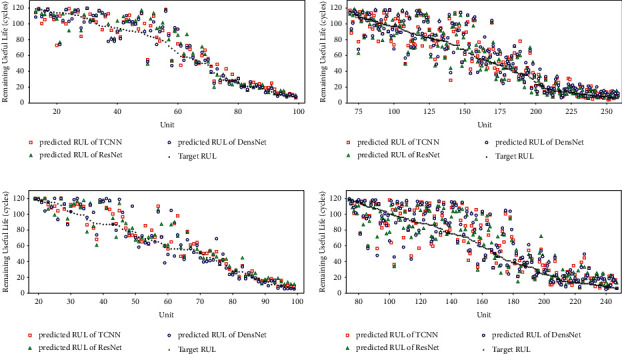
Comparison between the predicted RUL and the target RUL on test units. Units have been rearranged in decreasing order of the target RUL, and the units with an actual RUL higher than 120 are omitted. (a) FD001 test set. (b) FD002 test set. (c) FD003 test set. (d) FD004 test set.

**Figure 8:**
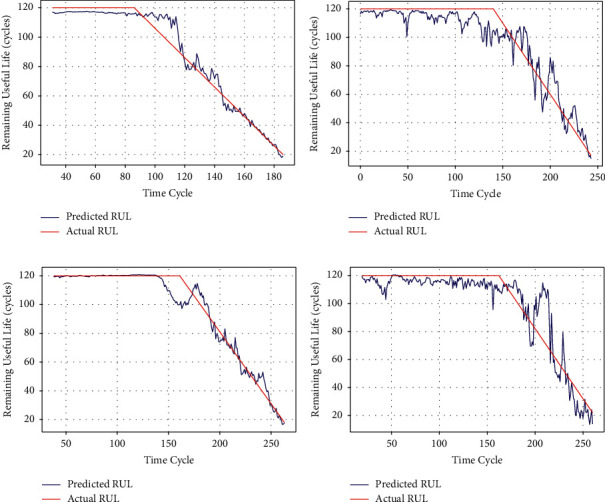
RUL predictions of units belonging to different subsets. (a) Unit 23 in FD001. (b) Unit 69 in FD002. (c) Unit 20 in FD003. (d) Unit 31 in FD004.

The experimental results of the proposed joint model using different CNN structures on the C-MAPSS dataset are shown in Figure 7. Figure 7 presents the RUL prediction results of the joint models on four subsets. Note that we rearrange the units of each test subset in descending order according to the target RUL (black dots in the figure). On the other hand, the middle and late stages of the unit life are of greater concern, so the units with an actual RUL higher than 120 are omitted in the figure to focus on the prediction results of units in the middle and late stages of degradation. As shown in Figure 7, for the units in the middle of the degradation process, the prediction results on the four subsets are not satisfactory, and many units have large prediction deviations. We believe that this is because the degradation features of these units are still not obvious enough. The recognition results of the model are somewhat ambiguous. Fortunately, the model shows high performance for the units in the late stage of the degradation process. We attribute this to the fact that the data of the units in the late stage of degradation contain more obvious fault information, and the model is more inclined to identify accurate fault patterns from these data. This characteristic is particularly important in practical industrial applications. Maintenance personnel can obtain more accurate RUL predictions at later stages of the system lifespan to avoid unexpected failures. We select units with relatively complete running cycles from the four test subsets and present their respective prediction results in Figure 8 as a supplementary explanation of the above characteristics. It can be seen that, for the entire lifespan of a single unit, as the unit degradation progresses, the closer to the failure point it is, the higher the prediction accuracy of the RUL is.

Examining the results of the models on the four datasets indicates that the prediction results of the models on FD001 and FD003 are much better than those of FD002 and FD004. From the prior knowledge from the C-MAPSS dataset, it can be inferred that this is due to the different complexity of subsets, which is manifested in the *operating conditions* and *fault modes*, i.e., FD001 contains a single operating condition and fault mode, while FD004 has the most complex case; it contains six operating conditions and two fault modes, which makes the prediction on FD004 particularly difficult. The operating conditions and fault modes of the datasets can be found in Table 4.

#### 4.3.2. Comparison of the Proposed Models

The evaluation metrics of the experimental results of the joint models on the C-MAPSS dataset are shown in Tables 6 and 7. The best results (except the last row) on each subset are shown in bold.  Table 6 corresponds to the RMSE metric described in equation (9), and Table 7 corresponds to the score metric described in equation (10). According to the experimental results of these two metrics, all the results of the joint model using TCNN on the three subsets, namely, FD001, FD002, and FD003, are the best, and the best result on the FD004 subset is obtained from ResNet. DenseNet's overall performance is slightly worse than TCNN and ResNet. This proved that the use of TCNN for 1D convolution in the time dimension is indeed slightly better than the use of ResNet and DenseNet.

**Figure 9:**
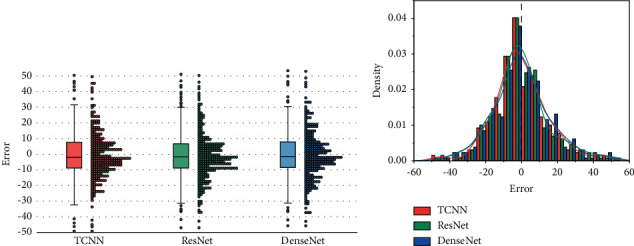
RUL prediction error distribution on the FD002 test set. (a) Box plot of prediction error. (b) Error histogram with the density curves.

In addition to the statistical metrics RMSE and score provided in Tables 6 and 7, respectively, Figure 9 gives the box plot and the histogram with the density curve (obtained by kernel density estimation) of the prediction error of the three models on the FD002 test set, which clearly and intuitively shows the overall situation of the prediction results of the models. In the box plot shown in Figure 9(a), the upper and lower edges of the box represent the upper and lower quartiles, and the whiskers show the position of the most extreme data point in the range of 1.5 times the quartile. We observed that the median and upper and lower quartiles of the three models are very close, but the joint model using TCNN has fewer outliers. Each outlier in the figure actually represents a bad prediction of RUL, so the joint model using TCNN has a more robust prediction result than the other two models and can obtain a relatively reliable prediction result.  Figure 9(b) shows the histograms of the RUL prediction error and its density curves. The three density curves are actually very close, and the peak positions of the three are very close to the ideal position. However, it can still be observed that TCNN is not prone to high underestimated life, while ResNet is not prone to high overestimated life.

**Figure 10:**
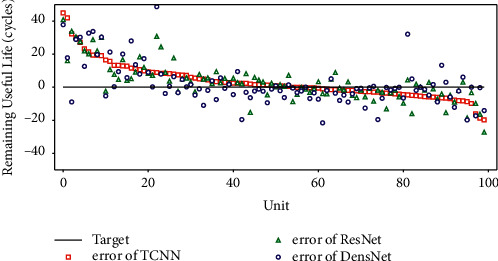
Comparison of prediction error on FD003.

RMSE is sensitive to large deviations. To evaluate the impact of large deviation values, the statistical metric mean absolute error (MAE) of the prediction results is shown in Table 8. The results in Table 8 show that there are some difficult-to-predict samples in the prediction results of the model. Interestingly, the MAEs of the three models on all subsets are very close. To verify whether the three models give similar results for each test sample, we rearrange the prediction errors of the three models according to the descending order of TCNN's prediction error (*e*_*i*_=RUL_*i*_^(prediction)^ − RUL_*i*_^(target)^). The results are shown in Figure 10. Only the results on FD003 are given here. Figure 10 shows that the prediction results of the joint models for a single test sample do not show a consistent trend, which proves that the three models learned different discrimination methods. We average the prediction results of the three models, which directly represents the prediction results of a simple ensemble learning model composed of three joint models. The corresponding RMSE metric can be found in the last row of Table 6. Compared with the single joint model, the ensemble model can further improve the prediction accuracy. However, the disadvantage of the ensemble model is that the time cost of training and prediction will increase several times, which increases the difficulty of online application. Therefore, we believe that a joint learning model such as “Transformer + TCNN” has more practical value.

#### 4.3.3. Comparison with Previous Work

Many previous works have achieved some results on the C-MAPSS dataset. To prove our research progress, we use the best “Trans. + TCNN” model to compare it with the previous research results. The comparison of RMSE is shown in Table 9, and the comparison of the score is shown in Table 10. The best results on each subset are still in bold. The last row of the table shows the improvement or retrogression of our model compared with the best results in the past.

As shown in Tables 9 and 10, our joint model has shown excellent performance and has made a comprehensive lead in the FD002 and FD004 subsets. Specifically, the most significant improvement occurred in the score metric on the FD002 subset, which improved 53.6% compared to the best result of the previous works, and the smallest improvement occurred in the RMSE metric on the FD004 subset, which increased by 17.8%, still a considerable number. Unfortunately, the performance of our joint model is degraded by 10.0% in the score metric of the FD003 subset. In addition, in the comparison of the remaining several results, our joint model has only slight improvement or retrogression. We have learned that, compared with the FD001 and FD003 subsets, the FD002 and FD004 subsets have more complex fault modes and operating conditions; therefore, our joint model is more robust on complex datasets and shows better performance, without significant degradation on simple datasets. This means that our model has indeed been successful.

## 5. Conclusion and Future Perspectives

In this work, we proposed a joint deep learning model combined with the transformer encoder and CNN for the RUL prediction task. We use the self-attention mechanism of the transformer to capture the cross-distance dependence in the time series and eliminate the distance limitation between historical data; therefore, the length of the input time series data does not affect the performance of the model. This is difficult for recurrent neural networks. Moreover, considering that the self-attention mechanism is not particularly sensitive to the local context and that the sensor data often have a strong local correlation, we use the CNN to extract local information. We compared three different structures of CNNs through experiments, and the results prove that TCNN using 1D convolution is more suitable for multivariate sensor time series data. The regression module makes our joint learning model different from the ensemble learning model, which is also composed of multiple models. The application of FRM enables the model to recalibrate the importance of the output features of multiple models. The experimental results on the C-MAPSS dataset show that the performance of our joint model is better than that of the previous work under complex fault modes and operating conditions. Our joint deep learning model for RUL prediction can be combined with different models to adapt to different tasks. It is flexible and has development potential.

There are several limitations here that deserve further study. First and most intuitive, the proposed approach has some performance degradation compared with previous works with simple operating conditions and fault modes. A reasonable conjecture is that the lack of structural bias in the transformer architecture makes it prone to overfitting on small-scale data (i.e., FD001 and FD003). How to further improve the generalization performance of the model is a direction worth discussing. Additionally, many fields tend to focus on the application of the RUL prediction algorithm in real-time online prediction, and our work does not evaluate the online prediction performance of the proposed approach. This requires the algorithm to balance time cost and accuracy. How to optimize the model to complete this is worthy of further research.

## Figures and Tables

**Table 1:** Recent deep architecture-based approaches for RUL prediction.

Authors	Year	Approach	Object
Hasani et al. [32]	2017	Autoencoder	Bearing
Li et al. [16]	2019	CNN	
Hu et al. [33]	2019	DBN	
Jiang et al. [34]	2020	Attention-LSTM	

Badu et al. [15]	2016	CNN	Turbofan
Zhang et al. [17]	2016	Multi-DBN	
Al-Dulaimi et al. [26]	2019	LSTM + CNN	
Liu et al. [31]	2019	Autoencoder	
Agrawal et al. [25]	2021	Optimized LSTM-GRU	

Li et al. [24]	2020	LSTM	Battery
Yu et al. [28]	2020	Bi-LSTM	
Ren et al. [35]	2021	CNN-LSTM	
Bhattacharya et al. [14]	2021	MFO-DNN	

**Table 2:** Hyperparameters determined by random search.

	Hyperparameter	Range	Type
1	Layers of encoder	[1, 6]	Discrete
2	Layers of TCNN	[1, 4]	Discrete
3	Kernel size of TCNN	[2, 5]	Discrete
4	Learning rate	[1*e* − 4, 1*e* − 1]	Continuous
5	Size of hidden layer of FCN	[16, 256]	Discrete
6	Size of hidden layer FFN	[16, 256]	Discrete
7	Batch size	[16, 128]	Discrete

**Table 3:** Complexity and parameter counts of core components of the proposed joint model. *d* is the sequence length. *k* is the representation dimension. *s* is the kernel size of convolutions.

Module	Complexity	Parameters (K)
Self-attention	*O*(*d* · *k*^2^)	17
FFN	*O*(*d*^2^ · *k*)	40
TCNN	*O*(*s* · *d* · *k*^2^)	53
ResNet	*O*(*s* · *d* · *k*^2^)	67
DenseNet	*O*(*s* · *d* · *k*^2^)	46

**Table 4:** Details of C-MAPSS dataset.

C-MAPSS	FD001	FD002	FD003	FD004
Min. cycles of TE	31	21	38	19
Number of units in TR	100	260	100	249
Number of units in TE	100	259	100	248
Operating conditions	1	6	1	6
Fault modes	1	1	2	2

^*∗*^TR: training subsets; TE: testing subsets.

**Table 5:** Chosen hyperparameter values.

	Hyperparameter	Value
1	Layers of encoder	3
2	Layers of TCNN	4
3	Kernel size of TCNN	3
4	Learning rate	1*e* − 4
5	Size of hidden layer of FCN	64
6	Size of hidden layer FFN	64
7	Batch size	32

**Table 6:** RMSE comparison of the joint models.

Architecture	FD001	FD002	FD003	FD004
Trans. + TCNN	**12.31**	**15.35**	**12.32**	18.35
Trans. + ResNet	12.75	15.58	12.46	**17.97**
Trans. + DenseNet	12.47	16.08	13.05	18.92
Ensemble of above	11.40	14.75	11.35	17.30

**Table 7:** Score comparison of the joint models.

Architecture	FD001	FD002	FD003	FD004
Trans. + TCNN	**252**	**1267**	**296**	2120
Trans. + ResNet	288	1280	318	**2079**
Trans. + DenseNet	254	1453	415	2583

**Table 8:** MAE comparison of the joint models.

Architecture	FD001	FD002	FD003	FD004
Trans. + TCNN	9.06	11.54	7.67	13.60
Trans. + ResNet	9.02	11.50	8.51	13.72
Trans. + DenseNet	9.06	12.08	8.58	13.78

**Table 9:** RMSE comparison of ours and prior methods.

Methods	FD001	FD002	FD003	FD004
CNN [15]	18.45	30.29	19.82	29.19
LSTM [52]	16.14	24.49	16.18	28.17
BiLSTM [53]	13.65	23.18	13.74	24.86
BiLSTM + MSCNN [29]	12.75	22.46	**11.35**	24.10
DAG network [54]	**11.96**	20.34	12.46	22.43
MHCNN + LSTM [55]	12.19	19.93	12.85	22.89
RULENet [56]	13.96	22.19	14.76	25.41
Trans. + TCNN	12.31	**15.35**	12.32	**18.35**

IMP	−2.9%	23.0%	−8.5%	17.8%

**Table 10:** Score comparison of ours and prior methods.

Methods	FD001	FD002	FD003	FD004
CNN [15]	1290	13600	16000	7890
LSTM [52]	338	4450	852	5550
BiLSTM [53]	295	4130	317	5430
BiLSTM + MSCNN [29]	281	5170	**278**	4790
DAG network [54]	**229**	2730	553	3370
MHCNN + LSTM [55]	259	4350	343	4340
RULENet [56]	310	3900	310	3800
Trans. + TCNN	252	**1267**	296	**2120**

IMP	−10%	53.6%	−6.5%	37.1%

## Data Availability

Previously reported C-MAPSS data were used to support this study and are available at https://ti.arc.nasa.gov/tech/dash/groups/pcoe/prognostic-data-repository/. The prior study (and dataset) is cited at the relevant place within the text as a reference [47].
